# Supplementary material for: Predicting visual function by interpreting a neuronal wiring diagram
Source: Nature. 2024 Oct 2;634(8032):113–23. doi: 10.1038/s41586-024-07953-5 (PMC11446822; doi:10.1038/s41586-024-07953-5)
Supplement: Supplementary file 6 — CRF and ERF predictions for individual TmY4 and TmY9 cells. Analogous to Supplementary Data 3, but for TmY target types. Shown are the top four monosynaptic pathways, the strongest pathway passing through each of the top ten intermediary types (ranking from Extended Data Fig. 7), and the trisynaptic pathway Tm1–TmY–Dm3–TmY (see the section entitled Prediction of spatial normalization). [file 41586_2024_7953_MOESM6_ESM.zip › DataS4/TmY9qΓèÑ/720575940631789260.pdf]

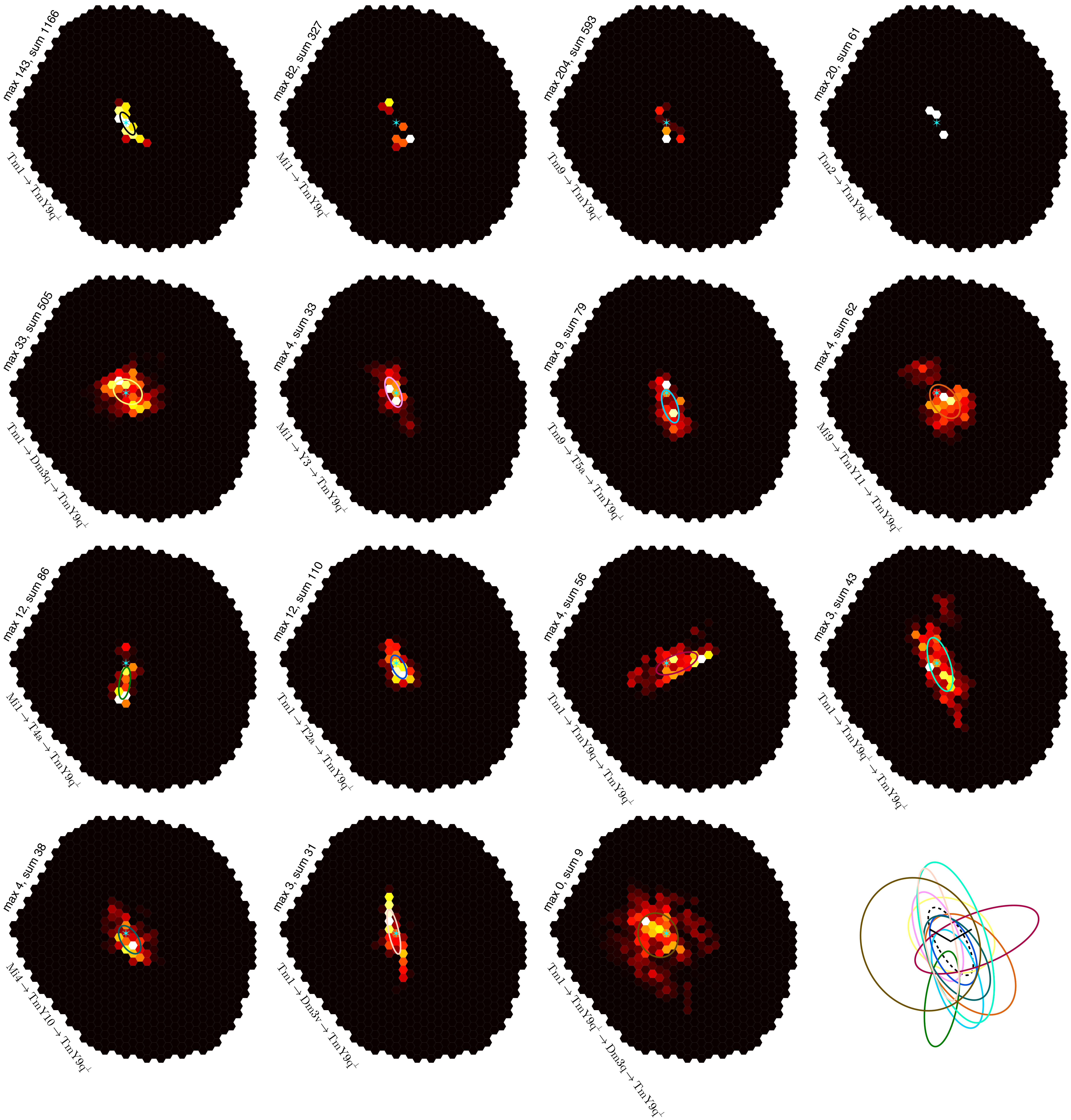
max 143, sum 1166
Tm1 → TmY9q⊥
max 82, sum 327
Mi1 → TmY9q⊥
max 204, sum 593
Tm9 → TmY9q⊥
max 20, sum 61
Tm2 → TmY9q⊥
max 33, sum 505
Tm1 → Dm3q → TmY9q⊥
max 4, sum 33
Mi1 → Y3 → TmY9q⊥
max 9, sum 79
Tm9 → T5a → TmY9q⊥
max 4, sum 62
Mi9 → TmY11 → TmY9q⊥
max 12, sum 86
Mi1 → T4a → TmY9q⊥
max 12, sum 110
Tm1 → T2a → TmY9q⊥
max 4, sum 56
Tm1 → TmY9q → TmY9q⊥
max 3, sum 43
Tm1 → TmY9q⊥ → TmY9q⊥
max 4, sum 38
Mi4 → TmY10 → TmY9q⊥
max 3, sum 31
Tm1 → Dm3v → TmY9q⊥
max 0, sum 9
Tm1 → TmY9q⊥ → Dm3q → TmY9q⊥
